# Factors affecting survival after liver retransplantation: a systematic review and meta-analysis

**DOI:** 10.3389/frtra.2023.1181770

**Published:** 2023-05-31

**Authors:** Elizabeth W. Brombosz, Linda W. Moore, Constance M. Mobley, Sudha Kodali, Ashish Saharia, Mark J. Hobeika, Ashton A. Connor, David W. Victor, Yee Lee Cheah, Caroline J. Simon, Ahmed Osama Gaber, Rafik Mark Ghobrial

**Affiliations:** ^1^ Department of Surgery, Houston Methodist Hospital, Houston, TX, United States; ^2^ Department of Surgery, Weill Cornell Medical College, New York, NY, United States; ^3^ JC Walter Jr Transplant Center, Houston Methodist Hospital, Houston, TX, United States; ^4^ Sherrie and Alan Conover Center for Liver Disease and Transplantation, Houston Methodist Hospital, Houston, TX, United States; ^5^ Department of Medicine, Weill Cornell Medical College, New York, NY, United States

**Keywords:** liver transplantation, liver retransplantation, meta-analysis, reoperation, end-stage liver disease, risk factors

## Abstract

**Background:**

Liver retransplantation (reLT) has historically had inferior survival relative to primary liver transplant (LT). To improve outcomes after reLT, researchers have identified factors predicting overall (OS) and/or graft survival (GS) after reLT. This systematic review and random effects meta-analysis sought to summarize this literature to elucidate the strongest independent predictors of post-reLT.

**Methods:**

A systematic review was conducted to identify manuscripts reporting factors affecting survival in multivariable Cox proportional hazards analyses. Papers with overlapping cohorts were excluded.

**Results:**

All 25 included studies were retrospective, and 15 (60%) were single-center studies. Patients on pre-transplant ventilation (HR, 3.11; 95% CI, 1.56–6.20; *p* = 0.001) and with high serum creatinine (HR, 1.46; 95% CI, 1.15–1.87; *p* = 0.002) had the highest mortality risk after reLT. Recipient age, Model for End-Stage Liver Disease score, donor age, and cold ischemia time >12 h also conferred a significant risk of post-reLT death (all *p* < 0.05). Factors affecting GS included donor age and retransplant interval (the time between LT and reLT; both *p* < 0.05). OS is significantly higher when the retransplant interval is ≤7 days relative to 8–30 days (*p* = 0.04).

**Conclusions:**

The meta-analysis was complicated by papers utilizing non-standardized cut-off values to group variables, which made between-study comparisons difficult. However, it did identify 7 variables that significantly impact survival after reLT, which could stimulate future research into improving post-reLT outcomes.

## Introduction

Liver retransplantation (reLT), also called “redo” liver transplantation or second liver transplantation, involves the replacement of a previously transplanted liver graft (usually orthotopically) with a new graft. It is a lifesaving procedure for approximately 2%–3% of liver transplant (LT) patients in the United States (1), 6.6% in Europe (2), 3% in Asia (3, 4), and 6.7% of patients in Australia (5). It is generally considered to be the only treatment option for patients with acute or chronic liver graft failure due to conditions such as primary nonfunction, hepatic artery thrombosis, or chronic rejection. However, reLT has been the subject of debate in the literature due to historically reduced survival relative to LT (6, 7). Authors have argued that reLT recipients should be carefully selected, as each liver graft allocated to reLT means another patient on the waitlist does not receive their LT (8). With advances in recipient and donor selection, both overall patient (OS) and graft (GS) survival have improved over time, approaching levels seen after primary LT (Table 1). Although reLT rates are low, the absolute numbers of reLTs performed are expected to increase as the number of transplant recipients grows.

**Table 1:** Direct comparisons of graft and overall survival rates after primary liver transplant (LT) and first liver retransplant (reLT) in adults.

Study	reLT Sample size	Graft survival rate						Overall survival rate					
		LT			reLT			LT			reLT		
		1-year	3-year	5-year	1-year	3-year	5-year	1-year	3-year	5-year	1-year	3-year	5-year
Tokat et al. 1995 (9)	96				43%			60%			73%		
Doyle et al. 1996 (6)	418	72.8%		59.5%	49.7%		35.5%						
Markmann et al. 1997 (7)	356							83%		74%	62%		47%
Pares et al. 1999 (10)	54				50%		45%				56%		50%
Bilbao et al. 2003 (11)	74							75.6%		64.8%	60.8%		49.5%
Watt et al. 2003 (12)	2,129							86%	79%	73%	67%	56%	52%
Ghabril et al. 2007 (13)	108	80%	72%		66%	62%		88%	82%		74%	70%	
Magee et al. 2007 (14)	2,372	83%	75%	69%	67%	60%	53%						
Yamauchi et al. 2007 (15)	36	77.1%		69.7%	56.6%		41.6%	77%		70%	62.6%		48.2%
Torres-Quevedo et al. 2009 (16)	79							83%	75%	69%	66%	52%	42%
Montenovo et al. 2014 (17)	2,710	85%		56%	71%		55%	88%		76%	75%		48%
Meneu Diaz et al. 2002 (18)	122							85%	83%	78%	62%	53%	46%
Immordino et al. 2014 (19)	48							63%	60%	57%	56%	53%	46%
Martí et al. 2014 (20)	26	82.7%	70.9%	64.3%	65.4%	46.2%	42.3%						
Al-Freah et al. 2017 (21)	150	83.5%	80.6%		72.6%	70.7%		88%	81%	69%	73%	71%	55%
Croome et al. 2019 (22)	275												
2002–2007	181	86.3%	79.5%	73.9%	74.0%	66.2%	59.7%			74.0%			70.8%
2013–2017	94	91.1%	82.7%		88.7%	84.2%							
Jeffrey et al. 2019 (5)	302				79%		69%				80%		72%
2001–2017	218	88%		79%	85%		75%	93%		83%	89%		81%
Takagi et al. 2020 (23)	336							84.5%	78.0%	74.0%	79.3%	74.3%	70.8%
Salimi et al. 2021 (24)	64							82%	80%	70%	59%	43%	32%

To improve the allocation of liver grafts for reLT, many groups have published studies elucidating prognostic factors for post-reLT survival. Initial reports focused on outcomes after retransplantation for all recipients (6, 7, 25, 26) and high-risk subgroups, such as patients with recurrent hepatitis C (27–29). These reports found that recipient age, serum creatinine, total bilirubin, and indication for reLT, as well as donor age, were strongly associated with OS and GS (7, 27, 29–31).

The purpose of this systematic review and meta-analysis was to compile different variables that affect survival after reLT. Our goal was to identify prognostic factors that are the most strongly associated with post-reLT OS and GS. Strength was defined by the number of papers that identified a factor as significant (≥3) and the degree of the effect (size of the hazard ratio). The results presented here identify future areas of research to help improve outcomes for patients undergoing reLT and ensure the ethical allocation of scarce liver grafts.

## Materials and methods

**Figure 1:**
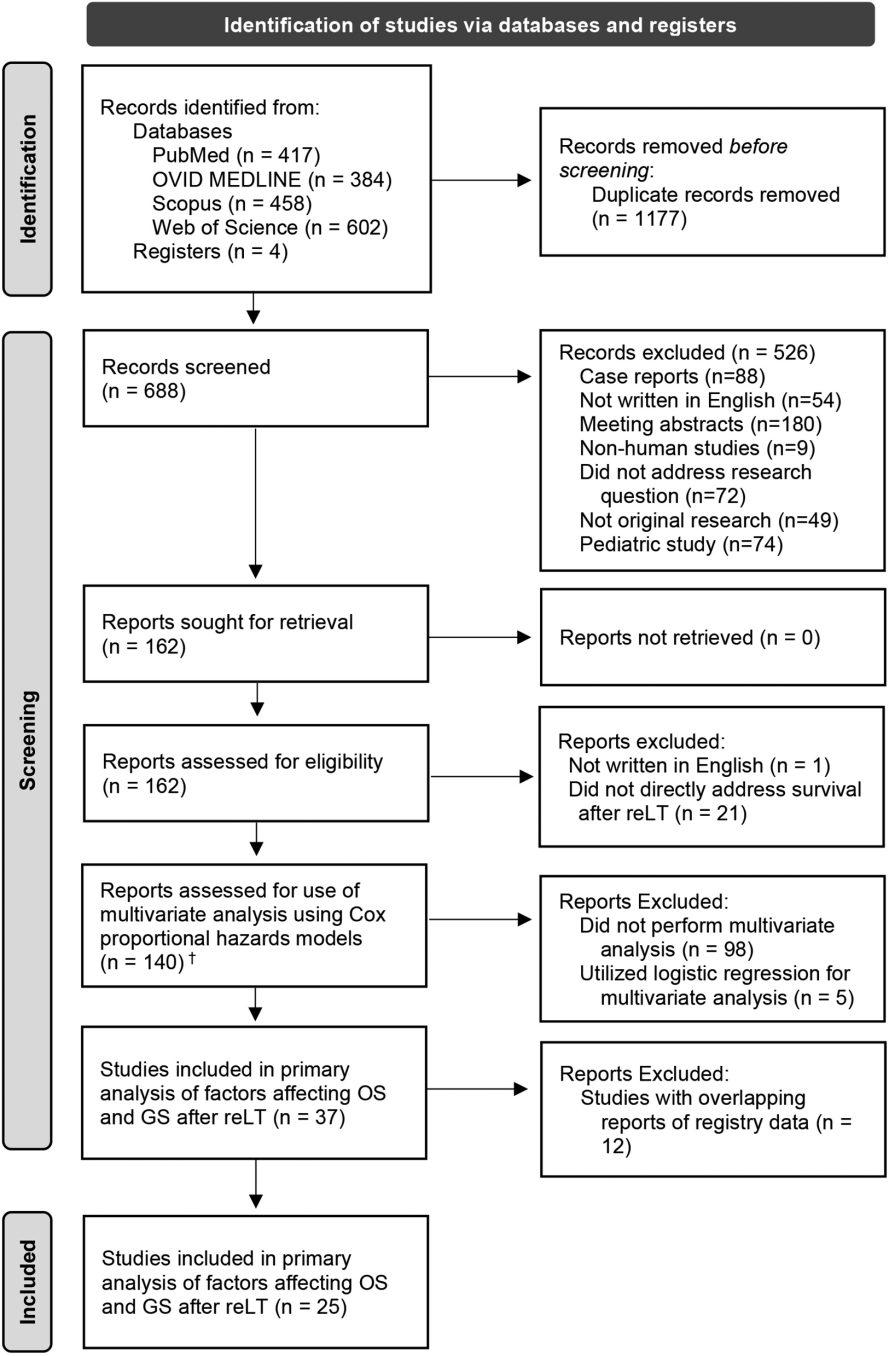
PRISMA flow diagram for the systematic review and meta-analysis of variables affecting overall survival (OS) and graft survival (OS) after liver retransplantation (reLT). ^†^These papers were further screened for analysis of the effects of retransplant interval on OS.

A systematic review of published literature was performed on March 23, 2022. The PRISMA diagram depicting procedures for identifying and screening records is presented in Figure 1. PubMed, OVID Medline, Scopus, Web of Science, and Cochrane Central were queried using the terms “liver retransplant*” and “liver re-transplant*” in separate searches. When possible, the search results were directly exported into comma-separated value or Excel files; otherwise, each result was manually entered into a spreadsheet. Duplicates were identified and removed to create a database of results for initial screening.

### Exclusion criteria and screening process

Case reports, studies exclusively reporting data on pediatric patients, studies not written in English, studies that did not include original data (e.g., reviews or letters to the editor), meeting abstracts and book chapters, studies on non-human subjects, studies that did not address factors affecting overall and/or graft survival after reLT, and studies that did not report the results of multivariable survival analysis were all excluded from the meta-analysis. The systematic review focused on outcomes in adult patients because more manuscripts addressed outcomes in adults (providing more data points for the meta-analysis). This also avoided confounding the analysis by including factors that may be relevant to pediatric but not adult outcomes.

The first screening step included reviewing the records (titles, abstracts, and journal titles) in the database search results. Full manuscripts were retrieved for all studies passing the first screen, which were subsequently screened to determine whether they met the inclusion/exclusion criteria.

### Meta-analysis of independent predictors of overall and graft survival after reLT

Papers reporting adjusted hazard ratios (HRs) were further analyzed for inclusion. HRs were included instead of ORs because most eligible studies utilized Cox proportional hazards analysis rather than logistic regression. Adjusted HRs were utilized instead of unadjusted (univariate) HRs to control for confounding factors. Only first reLTs were included; second reLT (re-reLT) and beyond were excluded. This analysis looked at both OS and GS, which was reported as the related outcome graft failure by some authors. In these instances, the HR was converted from graft failure risk to GS risk. Ultimately, 37 papers passed the first two screening steps.

For studies using national registry data, the most recent study was included, and all other studies were excluded from the meta-analysis for that variable. For example, when multiple studies were conducted using United States Scientific Registry of Transplant Recipients (SRTR) data, only the most recent paper was included in that analysis. Studies of subgroups of reLT recipients (e.g., hepatitis C virus-positive reLT recipients) were pooled with the other papers in the analysis unless there was a biological or medical rationale for excluding them. The one exception to this rule was the inclusion of data from a 2003 paper by Rosen et al., which reports data from SRTR, 6 centers in Europe, and 1 center in Australia (32). This paper was also included in meta-analyses when the dates studied in the paper (1986–1999) did not overlap with the most recent included studies (Supplementary Table S1). After removing papers with overlapping data sets, 25 studies remained. Quality of evidence was determined following Grading of Recommendations Assessment, Development, and Evaluation guidelines (33).

### Meta-analysis of the effects of the retransplant interval

Multiple studies have reported that the retransplant interval (the time interval between primary LT and reLT) has a significant effect on post-reLT outcomes (2, 7, 32, 34–36). In particular, many studies have reported that a retransplant interval of 0–7 or 0–10 days yields different survival rates from retransplant intervals of 7–30 or 10–30 days (5, 7, 37–40). However, most of the reports identified in the primary meta-analysis used different time cut-offs, making direct comparisons of HRs difficult.

Therefore, to better examine the effects of the retransplant interval on post-reLT survival, a second, independent meta-analysis was performed. This analysis screened eligible records (denoted by ^†^ in Figure 1) for reports of GS and/or OS relative to retransplant interval. Specifically, papers were included if they reported survival after retransplantation within the first 7–10 days after primary LT. Due to a lack of standardized cutoff times and definitions of “early” and “late” reLT, retransplant intervals >30 days were not included in the meta-analysis. The numbers of grafts/patients falling into each category (failed/no failure or deceased/alive) were compiled to perform the meta-analysis.

### Statistical analysis

When 3 or more studies reported HRs for the same variable, a forest plot was constructed, and a random effects meta-analysis was conducted utilizing that data (Table 2). Cochrane Review Manager (RevMan) 5.4.1 software (London, United Kingdom) was used to perform meta-analyses utilizing DerSimonian and Laird (41) random-effects models, and to construct forest plots. In the plots, studies were arranged chronologically to allow the examination of possible changes in effect sizes over time, although this was not a primary goal of the analysis. *p*-values ≤0.05 were considered statistically significant.

**Table 2:** Factors affecting outcomes after liver retransplantation selected for inclusion in the meta-analysis.

Factors affecting overall survival
Recipient demographic variables
Age
Sex
Recipient laboratory variables
Serum creatinine
Bilirubin
Recipient severity of illness
Model for end-stage liver disease (MELD) score
Pre-retransplantation mechanical ventilation
Pre-retransplantation dialysis
Donor variables
Age
Cold ischemia time
Retransplant interval
Factors affecting graft survival
Donor variables
Age
Retransplant interval

## Results

Study characteristics for the 25 studies included in the main meta-analysis are presented in Supplementary Table S1. Papers were published between 1999 and 2021, covering patients transplanted between 1982 and 2019. Study cohorts encompassed patients transplanted over a median of 12 years (IQR, 9–18 years). The median study sample size of the studies was 135 (IQR, 48–213). All were retrospective studies. There were 15 (60%) single-center studies, 4 (16%) multicenter studies, and 6 (24%) studies incorporating one or more national databases. The overall quality of evidence presented in these papers was low to moderate. The retrospective nature of the studies downgraded quality, although limiting the meta-analysis to include multivariable analyses helped to control for potential confounding factors.

### Recipient demographic and laboratory variables and OS after reLT

**Figure 2:**
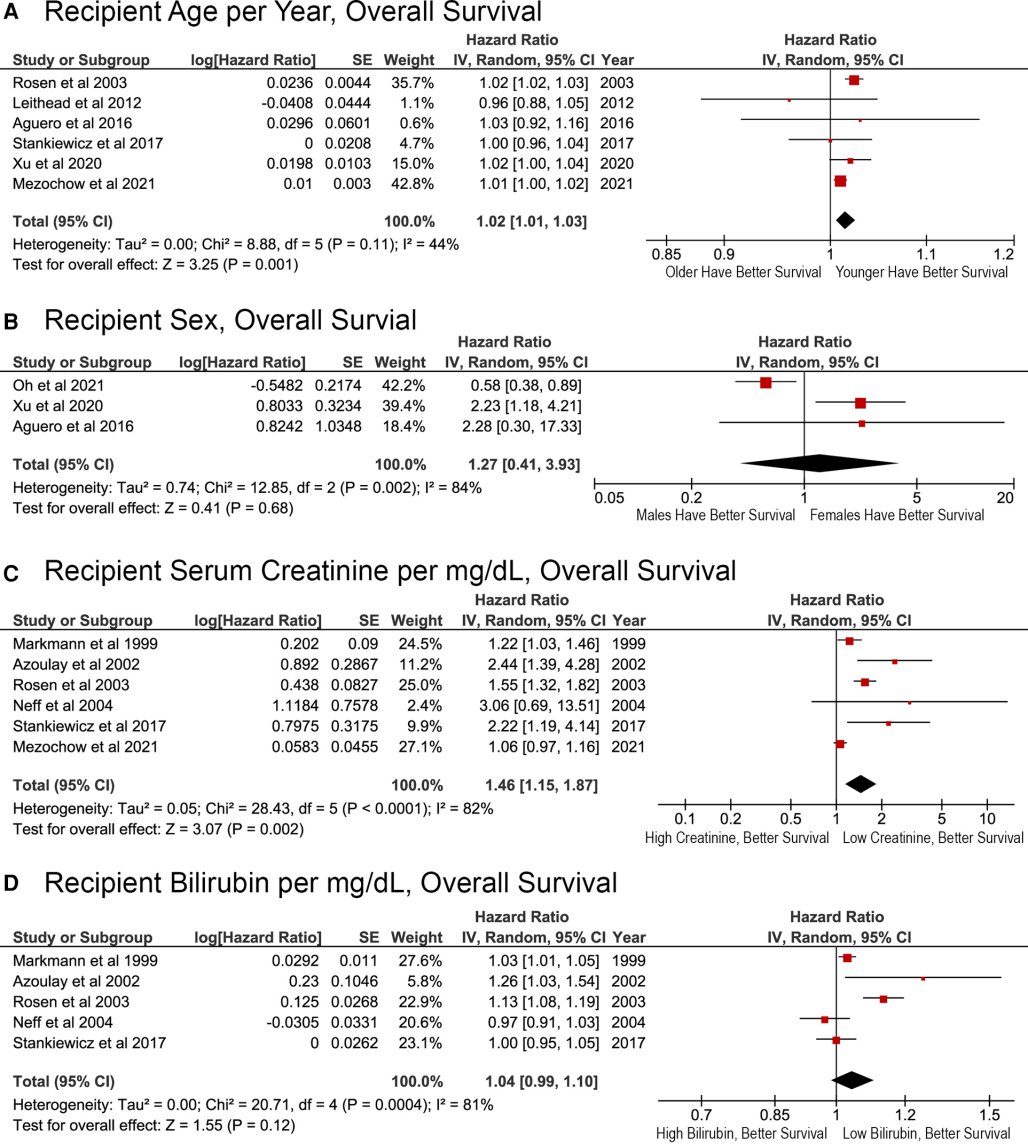
Effects of recipient demographic and pre-transplant laboratory variables on overall survival after liver retransplant. (**A**) Age per year; (**B**) sex, male or female; (**C**) pre-transplant serum creatinine per mg/dl; (**D**) pre-transplant total bilirubin per mg/dl.

Greater recipient age was significantly associated with reduced survival (*p* = 0.001), with a calculated HR of 1.02 (95% CI, 1.01–1.03) per year (Figure 2A). Recipient sex did not have a significant association with OS (HR, 1.27; 95% CI: 0.41–3.93; *p* = 0.68; Figure 2B). The ability of laboratory biomarkers to predict survival was mixed. Higher serum creatinine levels as a continuous variable were associated with worse survival (HR, 1.46; 95% CI, 1.15–1.87; *p* = 0.002; Figure 2C). Conversely, recipient total bilirubin levels were not significant associated with OS in the meta-analysis (HR, 1.04; 95% CI, 0.99–1.10; *p* = 0.12; Figure 2D). Although papers published in 2003 or earlier reported a significant association with bilirubin levels on OS, later papers did not (Figure 2D).

### Recipient severity of illness and OS after reLT

**Figure 3:**
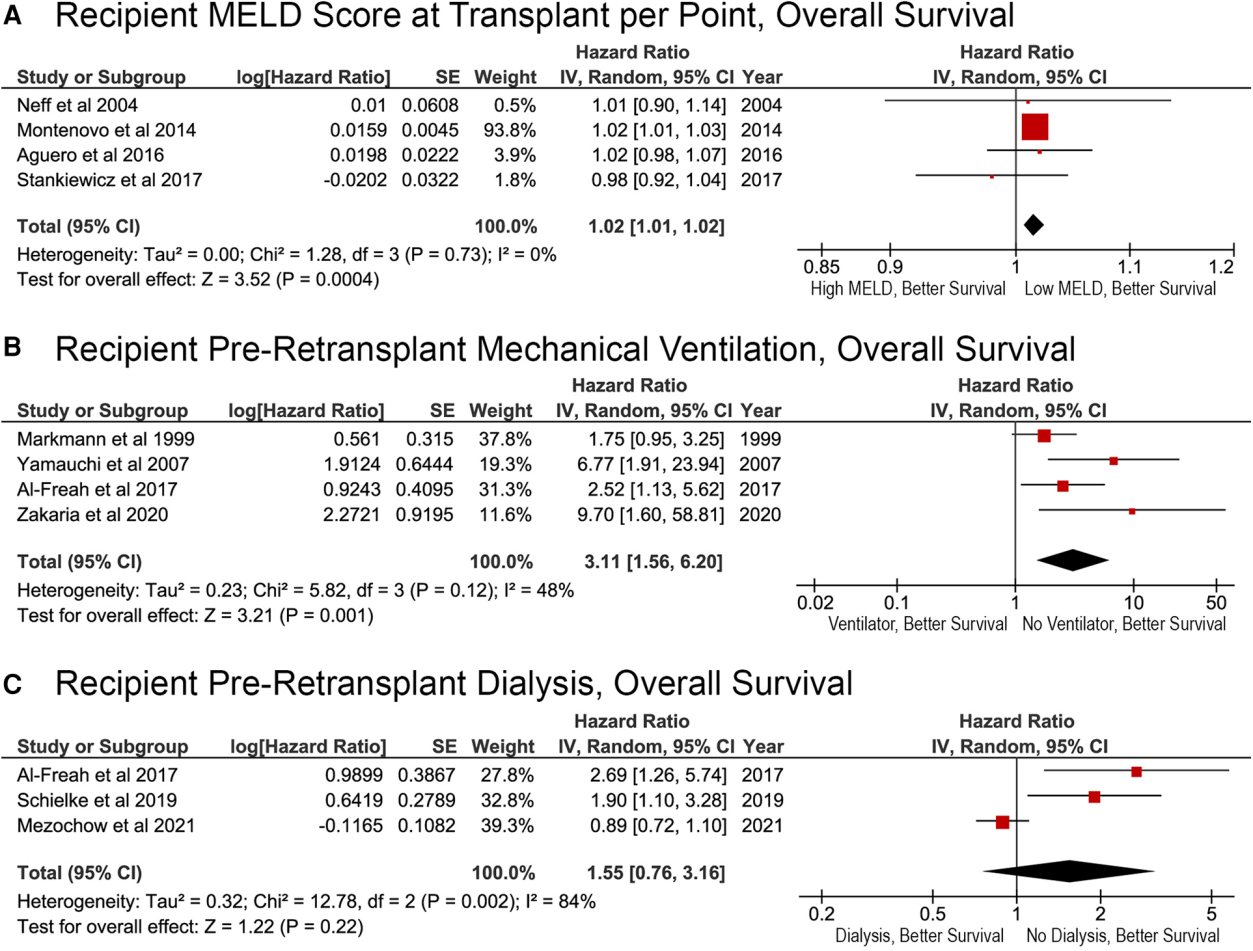
Effects of recipient severity of illness on overall survival after liver retransplantation. (**A**) Pre-transplant Model for End-Stage Liver Disease score, per point; (**B**) pre-retransplant mechanical ventilation status (yes or no); (**C**) pre-retransplant dialysis (yes or no).

Factors associated with recipient severity of illness were associated with reduced survival after reLT. Each additional MELD score point resulted in a significantly higher risk of death (HR, 1.02; 95% CI, 1.01–1.02; *p* = 0.0004; Figure 3A). Patients who were on mechanical ventilation before reLT were also at an increased risk of death (HR, 3.11; 95% CI, 1.56–6.20; *p* = 0.001; Figure 3B). Whether the recipient was on dialysis prior to reLT was not associated with OS (HR, 1.55; 95% CI, 0.76–3.16; *p* = 0.22; Figure 3C). The effect of pre-reLT dialysis has decreased over time and was not a significant factor in the most recent study (Figure 3C).

### Donor factors affecting OS after reLT

**Figure 4:**
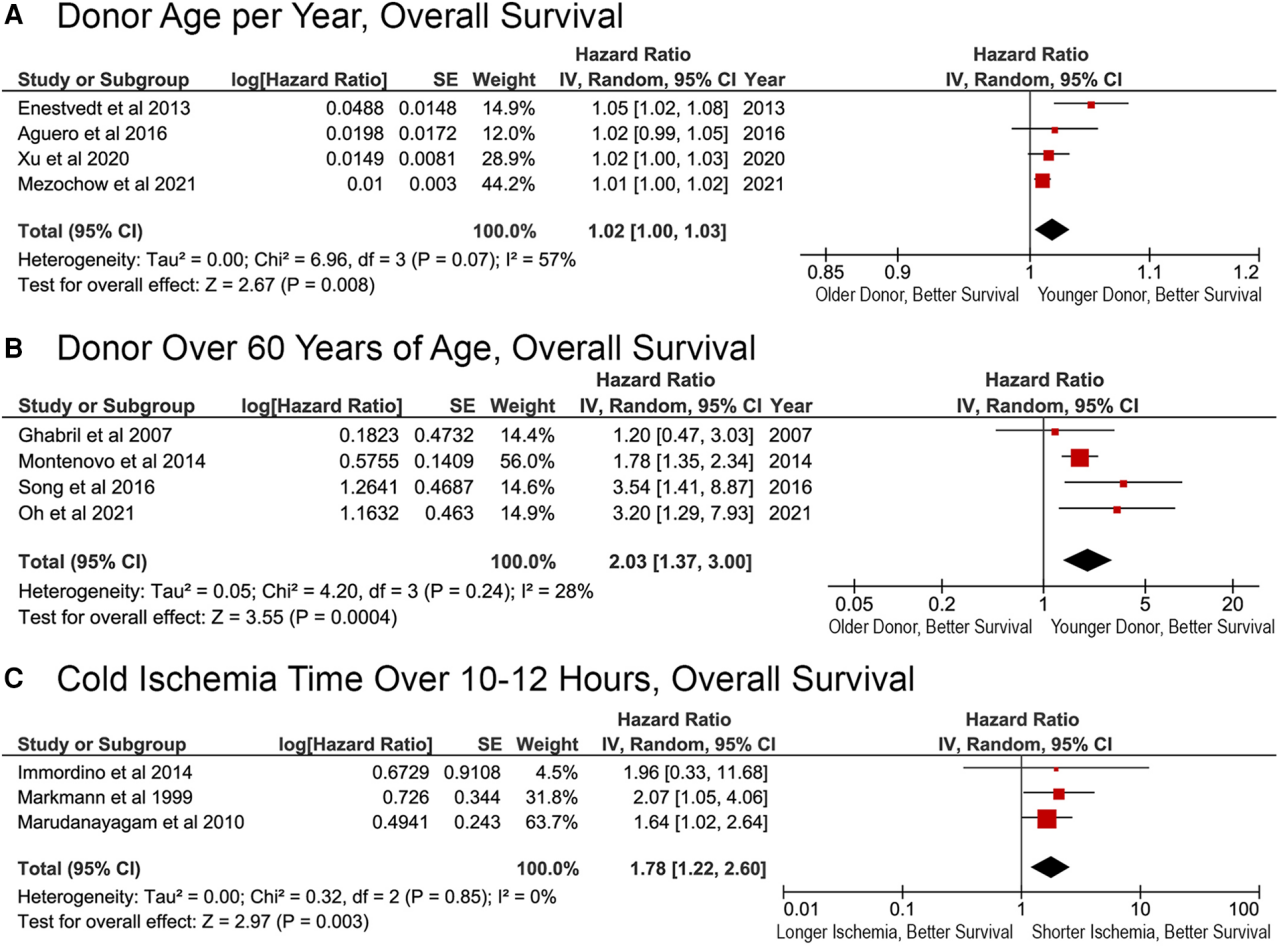
Effects of donor factors on overall survival after liver retransplantation. Effect of (**A**) donor age per year; (**B**) donor age >60 years; (**C**) cold ischemia time longer than 10–12 h.

Donor age was significantly associated with OS, both when considered as a continuous and categorical variable in the original Cox proportional hazards analysis. Meta-analysis revealed an overall HR of 1.02 per year (95% CI, 1.00–1.03; *p* = 0.008; Figure 4A). Donor age >60 years was also significantly related to OS (HR, 2.03; 95% CI, 1.37–3.00; *p* = 0.0004; Figure 4B). Cold ischemia time (CIT) of more than 10–12 h resulted in a significantly greater risk of death (HR, 1.78; 95% CI, 1.22–2.60; *p* = 0.003; Figure 4C).

**Figure 5:**
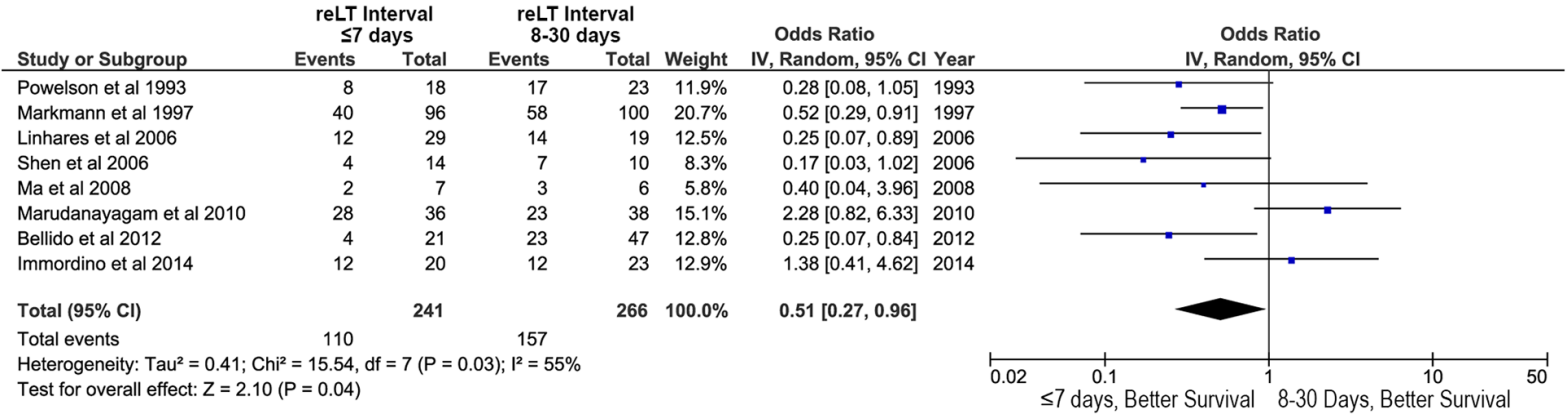
Effects of retransplant interval on overall survival after liver retransplantation: retransplantation within the first week after primary liver transplant (retransplant interval ≤7 days) vs. retransplantation between 8 and 30 days after primary liver transplant (retransplant interval 8–30 days).

### Retransplant interval and OS after reLT

Retransplant interval (the time between primary LT and reLT) was also associated with OS. A reLT within the first week after LT was associated with improved OS relative to reLT between days 8 and 30 after LT (OR, 0.51; 95% CI, 0.27–0.96; *p* = 0.04; Figure 5).

### Factors affecting GS after reLT

**Figure 6:**
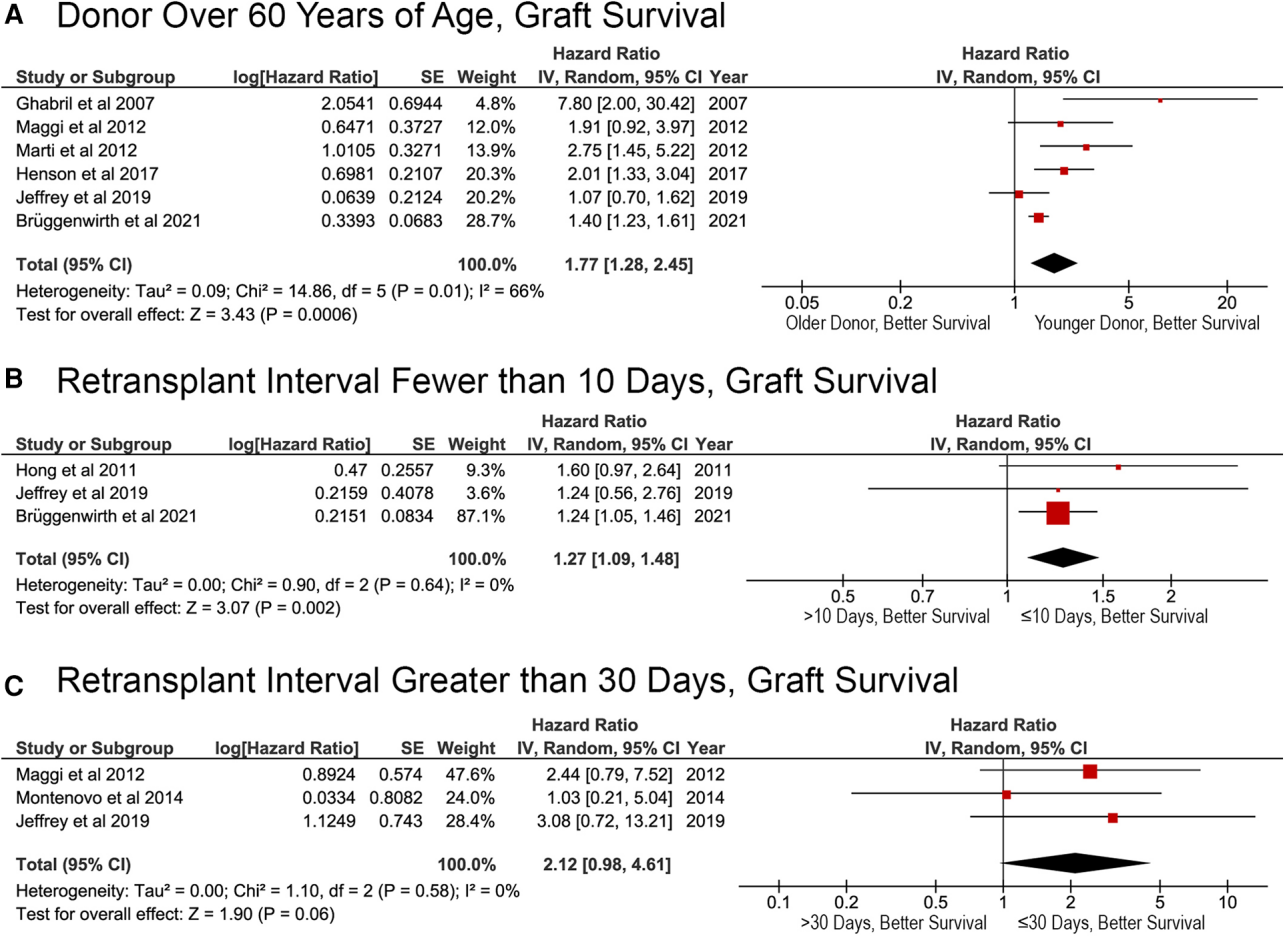
Factors affecting graft survival after liver retransplantation. (**A**) Donor age >60 years; (**B**) liver retransplantation within 10 days of primary liver retransplant (retransplant interval ≤10 or >10 days); (**C**) liver retransplantation within 30 days of primary liver retransplant (retransplant interval ≤30 or >30 days).

Fewer papers reported the results of multivariable analysis of the factors affecting GS after reLT, which limited the ability to perform the meta-analysis. However, two major risk factors did emerge: donor age and intermediate retransplant interval. Receiving a liver graft from a donor over the age of 60 was associated with a higher risk of death (HR, 1.77; 95% CI, 1.28–2.45; *p* = 0.0006; Figure 6A). Patients with a retransplant intervals of greater than 10 days were at a greater risk of death after reLT (HR, 1.27; 95% CI, 1.09–1.48; *p* = 0.002; Figure 6B). The effects of a retransplant interval of >30 days approached, but did not meet, statistical significance (HR, 2.12; 95% CI: 0.98–4.61; *p* = 0.06; Figure 6C).

## Discussion

The meta-analysis showed that multiple factors affect OS and GS after reLT across the literature. These factors can be used to carefully select patients for reLT and identify patients who are at the highest risk of graft failure or death after reLT for closer monitoring of complications and/or prophylactic treatments. Additional research into ameliorating the effects of these variables may improve outcomes for LT recipients who require reLT.

Preoperative and postoperative care have evolved greatly over the course of the included studies, which contain data on patients transplanted between 1982 and 2019 (Supplementary Table S1). In most cases, effect sizes did not change over time. However, there did appear to be a decrease in the mortality risk conferred by pre-reLT dialysis over time. There was a declining trend in the impact of increased donor age on OS and GS over time as well. Together, these trends suggest that improved medical care, including donor management, has ameliorated some risks of reLT.

### Recipient factors affecting survival after reLT

We found that recipient age, serum creatinine, MELD at LT, and pre-reLT mechanical ventilation significantly impacted overall survival. In general, older LT recipients have lower OS after transplantation, most frequently dying from non-transplant-related causes (42). Recipient MELD was also an important factor, with a 2% increase in mortality risk per MELD point increase. Other studies that did meet the inclusion for criteria for the MELD meta-analysis have also shown that patients with higher MELD scores have a poorer prognosis after reLT (2, 13, 37, 43–45). High-MELD patients requiring urgent reLT should be carefully evaluated and monitored postoperatively.

The meta-analysis shows that sicker patients with worse kidney, liver, and lung function are also more likely to die after reLT. Pre-reLT mechanical ventilation was the strongest predictor of OS in our analysis. Very sick patients with multiple failing organs are at a higher risk of post-LT mortality, and may even become “too sick” to transplant (46). These results imply that reLT recipients should be carefully selected to minimize so-called “futile” retransplants (8). The improvements in post-reLT survival trends over time (Table 1) suggest that transplant centers are continually enhancing outcomes for these patients, reducing the risk of a futile reLT.

### Donor factors affecting survival after reLT

This meta-analysis highlights the need for proper donor selection for reLT. Utilizing a graft from a donor over the age of 60 roughly doubled the recipient's risk of dying after reLT, an effect that was consistent throughout the included studies. Therefore, transplant teams should carefully weigh the risks and benefits of utilizing a liver graft from an older donor in reLT. Older donors are associated with an increased risk to LT recipients in general (47), although these risks can be mitigated by carefully selecting recipients for those grafts (48).

Extended CIT was also associated with poor outcomes after reLT. Longer CITs increase the risk of ischemia-reperfusion injury (49) and primary nonfunction (50). Our meta-analysis did not include donation after circulatory death graft recipients because there were too few papers reporting their effects in non-overlapping cohorts. However, individual studies have suggested that these donors may confer additional risk to patients undergoing reLT, in part due to extended CIT (51). Machine perfusion technology, which shortens CIT, will certainly affect future reLT outcomes (52). Perfusion has the potential to ameliorate the deleterious effects of long CIT and expand the number of grafts suitable for reLT.

### Retransplant interval and survival after reLT

The timing of the reLT operation can also have a strong effect on survival outcomes, with better OS and GS when reLT occurs within 7–10 days of LT. Researchers have argued that early reLT requires less effort during hepatectomy, shortening operative time and reducing blood loss (53). Additionally, a long reLT interval can extend the duration of multiple organ dysfunction caused by a failing liver graft, reducing the patient's chances of survival (6). Thus, the timely identification of patients who may need reLT is paramount. The importance of rapid reLT in patients with early graft failure implies that patients living in regions with longer wait times may be at a disadvantage (54).

### Limitations and sensitivity

The conclusions of this meta-analysis are limited by the quality of the papers included in the study. All included studies were retrospective in nature, and 60% were single-center studies. Six studies utilized registry data (Supplementary Table S1), which generally lack granularity and can have many missing values. In addition, the studies adjusted for different covariates in their multivariable models, which can affect results.

This analysis is also limited by the way that previous authors have chosen to analyze their data, which would also affect the sensitivity of the analysis. Many papers reported different cut-off values for categorical variables, and the results of many studies could not be compared directly. For example, some studies analyzing retransplant interval used a cutoff of 7 days to distinguish “early” vs. “late” reLT, while others used 10, 30, or even 90 days. It is possible that the effect sizes may have been over- or underestimated due to the way authors reported their data, which led to the exclusion of some studies. Funnel plots did not reveal any systematic publication biases across meta-analyses (Supplementary Figures S1–S4). However, the small number of studies in some comparisons make it difficult to draw strong conclusions regarding bias.

This analysis showed a high amount of heterogeneity (*I*^2^) in the effects of recipient sex, recipient serum creatinine, recipient total bilirubin, and recipient dialysis. These effects were likely due to large differences in sample sizes (Supplementary Table S1). Differences in the effect size at different centers and among different regions of the world may have also contributed to heterogeneity.

### Future areas of research

This systematic review and meta-analysis elucidated several potential areas for future research. First, studies should examine how to improve post-reLT outcomes for higher risk recipients, including ways to mitigate risk factors. Future research should also address the most frequent causes of morbidity and mortality in at-risk reLT recipients. Early treatments for potential postoperative graft injuries could lead to improved GS rates and improved patient health. In addition, machine perfusion technology has shown great promise for reducing CIT and ischemic-type biliary injuries, which can necessitate reLT (52, 55). Future work should elucidate how machine perfusion at LT affects the risk of needing reLT and how it might be utilized to improve the quality of extended-criteria grafts so they can be utilized in reLT.

## Conclusions

Previously published studies have identified dozens of variables that may affect OS and/or GS after reLT. Here, we sought to identify the strongest predictors of post-reLT outcomes. This systematic review and meta-analysis showed that recipient age, MELD score, serum creatinine levels, and mechanical ventilation status all significantly affect OS after reLT. In addition, recipients of liver grafts from older donors have a higher risk of post-reLT death, as do recipients with extended CIT during transplant. Donor age and the time between LT and reLT (retransplant interval) have a significant effect on GS. Retransplant within the first week after reLT results in improved survival relative to retransplant 8–30 days after LT. Although the meta-analysis was limited by the quality of data reported in the literature, it still identified important factors that affect survival after reLT.

## Data Availability

The data analyzed in this study is subject to the following licenses/restrictions: access to published peer-reviewed articles utilized in the meta-analysis requires personal or institutional subscriptions to the journals in which the articles are published. Requests to access these datasets should be directed to Elizabeth Brombosz, ewbrombosz@houstonmethodist.org.
