# Supplementary material for: Factors affecting survival after liver retransplantation: a systematic review and meta-analysis
Source: Front Transplant. 2023 May 31;2:1181770. doi: 10.3389/frtra.2023.1181770 (PMC11235252; doi:10.3389/frtra.2023.1181770)
Supplement: Supplementary file 1 [file Datasheet1.pdf]

Table S1. Studies included in the primary meta-analysis.

Paper	Year	n	Start Year	End Year	Location	Study Characteristics
Aguero et al 2016	2016	37	1997	2001	Spain, US, Italy, Germany, UK, Argentina, Portugal, Switzerland	Retrospective, multiple national databases
Al-Freah et al 2017	2017	150	2000	2007	UK (England)	Retrospective, single center
Azoulay et al 2002	2002	139	1986	2000	France	Retrospective, single center
Brüggenwirth et al 2021	2021	5581	2006	2016	Europe (ELTR data)	Retrospective, multinational data
Enestvedt et al 2013	2013	104	2000	2010	USA (Pennsylvania)	Retrospective, single center
Ghabril et al 2007	2007	60	1998	2004	USA (Florida)	Retrospective, single center
Henson et al 2017	2017	899	1987	2015	USA (SRTR data)	Retrospective, national database
Immordino et al 2014	2014	48	1988	2018	Italy	Retrospective, single center
Jeffrey et al 2019	2019	218	1986	2017	Australia, New Zealand	Retrospective, multinational database
Leithead et al 2012	2012	36	1994	2010	UK (England)	Retrospective, single center
Maggi et al 2012	2012	184	1998	2008	Italy (8 transplant centers)	Retrospective, single center
Markmann et al 1999	1999	150	1992	1996	USA (California)	Retrospective, single center
Marti et al 2012	2012	80	1988	2006	Spain	Retrospective, single center
Marudanayagam et al 2010	2010	196	1982	2007	UK (England)	Retrospective, single center
Mezochow et al 2021	2021	3483	2002	2018	USA (SRTR data)	Retrospective, national database
Montenovo et al 2014	2014	2710	2002	2012	USA (SRTR data)	Retrospective, national database
Neff et al 2004	2004	22	1996	2004	USA (Ohio, Florida)	Retrospective, dual center
Oh et al 2021	2021	258	2007	2016	South Korea (national database)	Retrospective, national database
Rosen et al 2003	2003	281	1986	1999	Germany, UK, Spain, Australia	Retrospective, multinational
Schielke et al 2019	2019	143	1987	2011	France (single center)	Retrospective, single center
Song et al 2016	2016	108	1994	2012	France, Spain (8 centers)	Retrospective, multicenter
Stankiewicz et al 2017	2017	49	not reported		Poland (single center)	Retrospective, single center
Xu et al 2020	2020	131	1991	2018	Canada (single center)	Retrospective, single center
Yamauchi et al 2007	2007	41	1990	2002	Japan (single center)	Retrospective, single center
Zakaria et al 2020	2020	32	2011	2019	Saudi Arabia (single center)	Retrospective, single center

ELTR, European Liver Transplant Registry; SRTR, Scientific Registry of Transplant Recipients; UK, United Kingdom; USA, United States of America

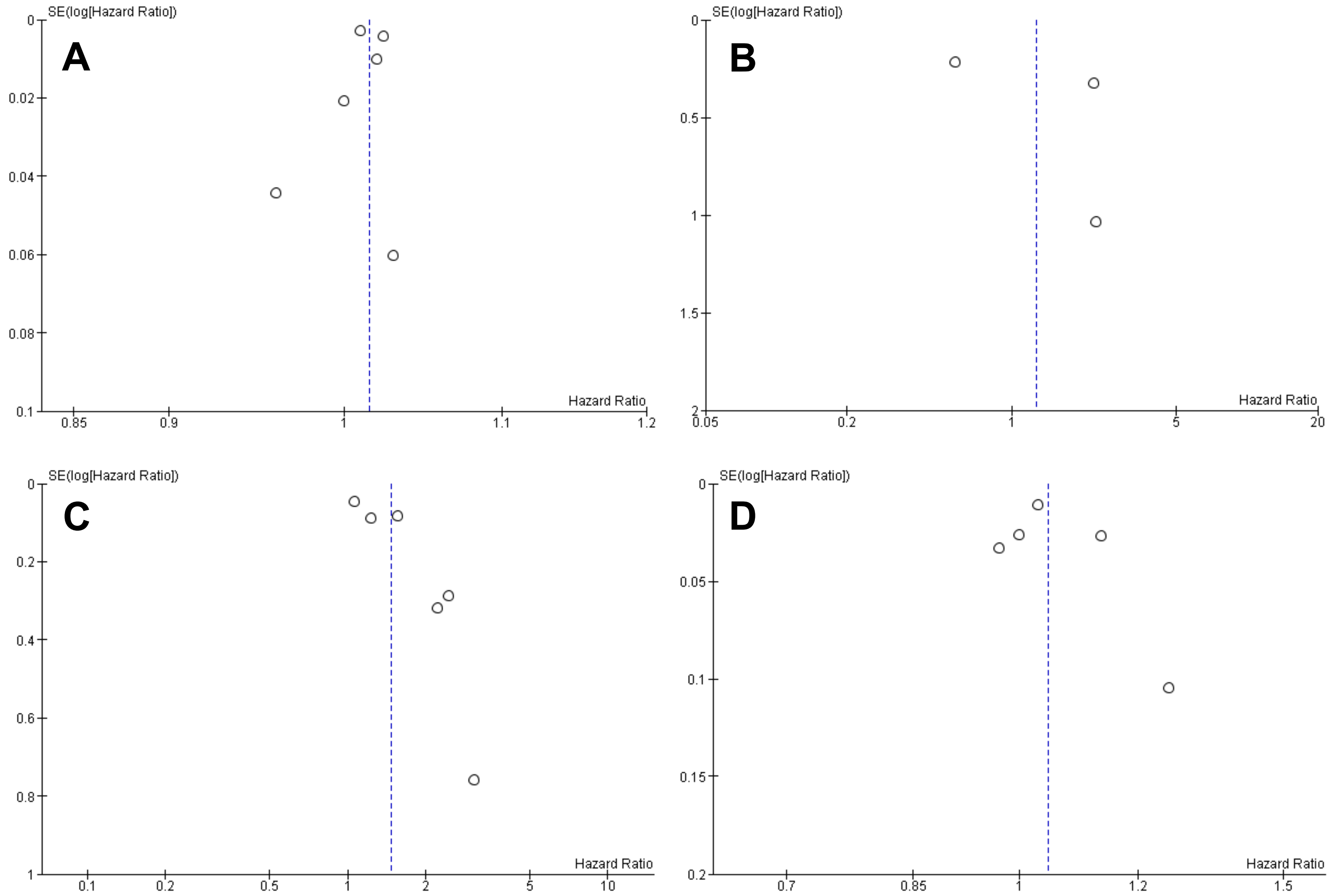

Figure S1. Funnel plots for the effects of (A) recipient age, per year. (B) recipient sex, (C) pre-transplant serum creatinine, and (D) pre-transplant bilirubin on overall survival after liver retransplant.

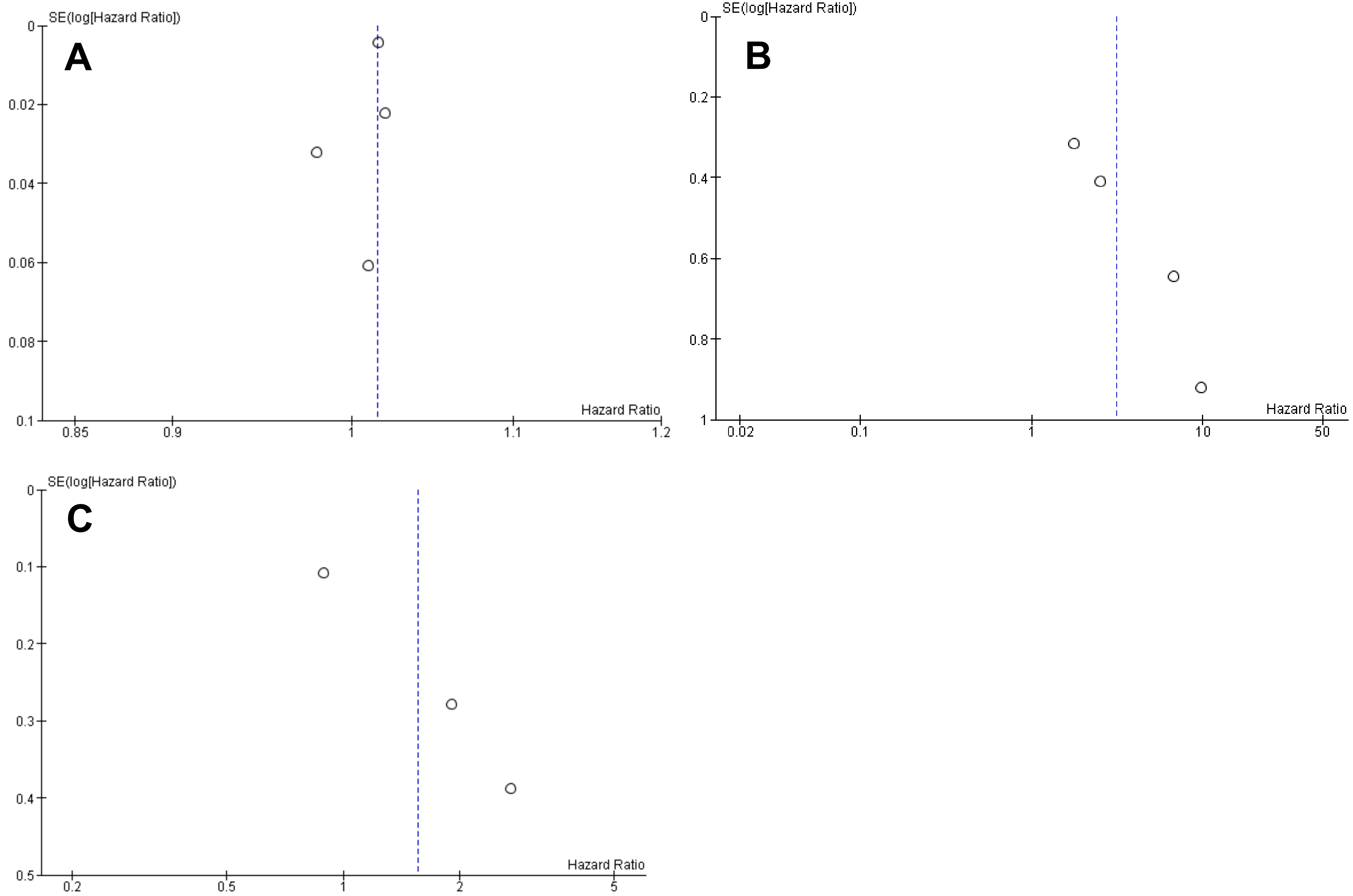

Figure S2. Funnel plots for (A) pre-transplant Model for End-Stage Liver Disease Score, (B) pre-retransplant mechanical ventilation status (on ventilator or not), and (C) of pre-retransplant dialysis (recipient on dialysis prior to retransplant or not) on overall survival after liver retransplant.

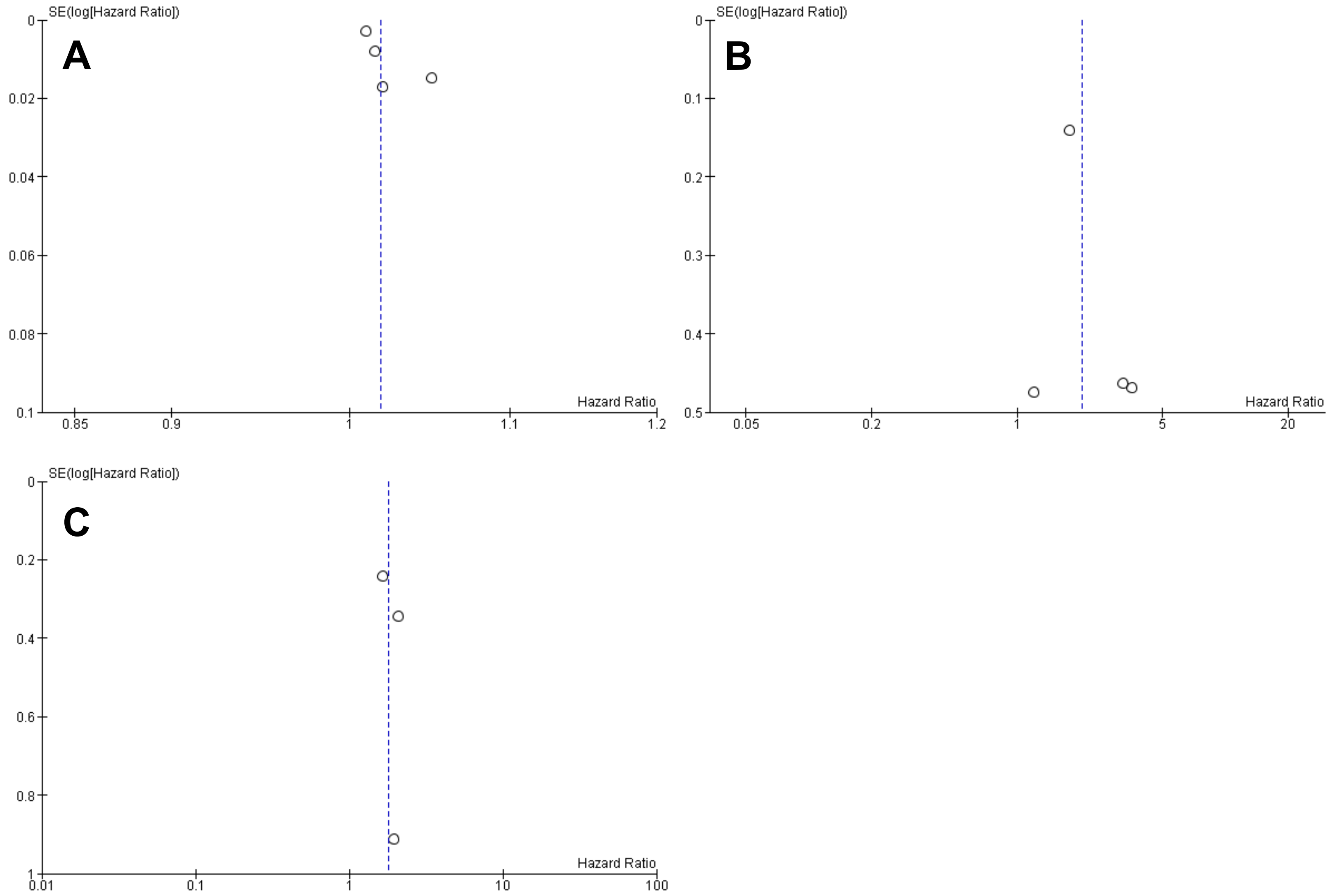

Figure S3. Funnel plots for (A) donor age (per year), (B) donor age >60 years, and (C) cold ischemia time longer than 10-12 hours on overall survival after liver retransplant.

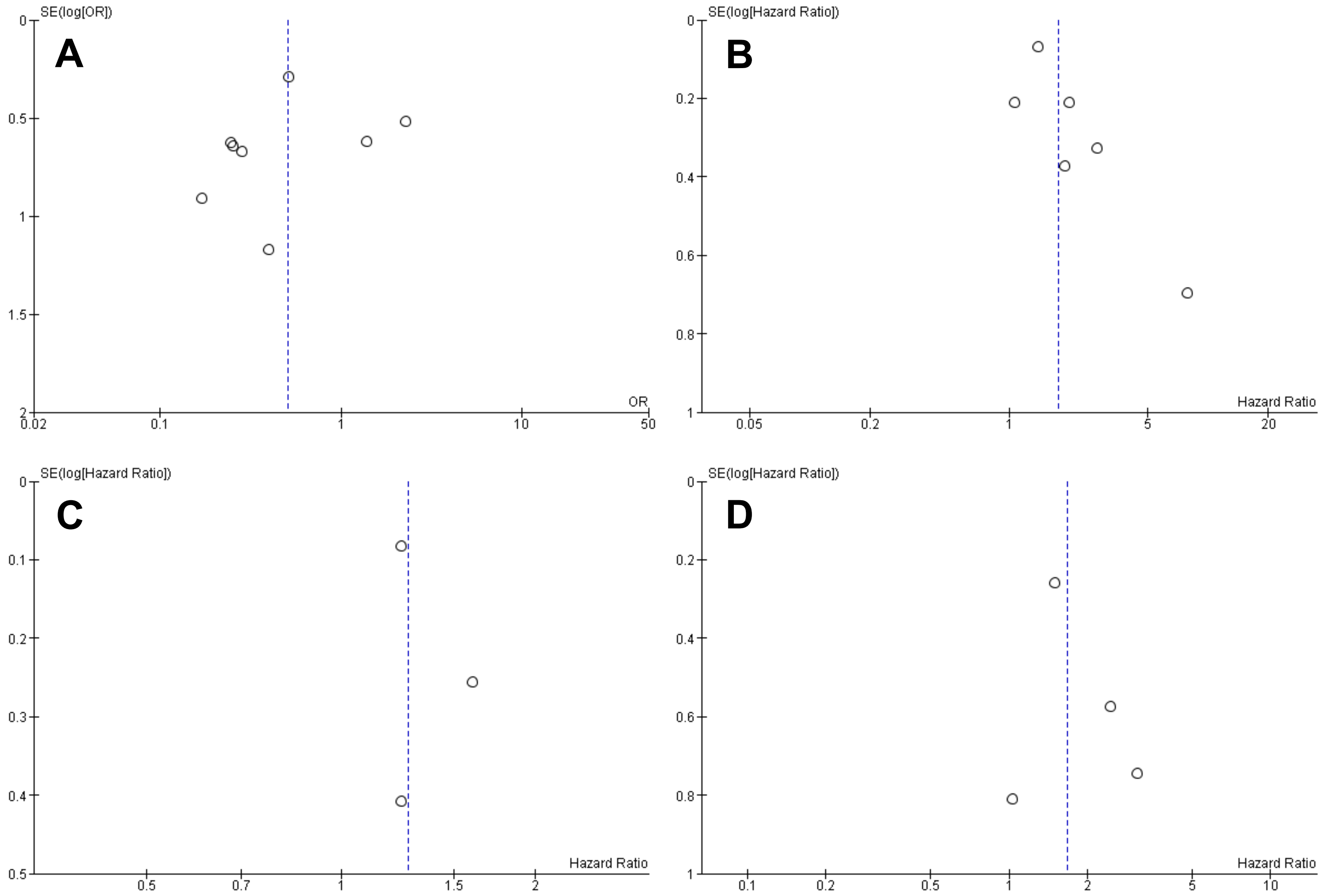

Figure S4. Funnel plots for (A) overall survival after retransplant interval of ≤7 days vs 8-30 days, and graft survival after (B) receiving a graft from a donor >60 years of age, (C) retransplant interval <10 days, and (D) retransplant interval >30 days).
